# Saccadometry Findings in Migraine Patients Without Aura

**DOI:** 10.1002/brb3.70367

**Published:** 2025-02-28

**Authors:** Asya Fatma Men, Aysenur Kucuk Ceyhan, Mustafa Men, Reyhan Sürmeli

**Affiliations:** ^1^ Department of Audiology Faculty of Health Sciences University of Health Sciences Istanbul Turkey; ^2^ Department of Neurology Ümraniye Education and Research Hospital Istanbul Turkey

**Keywords:** cognitive impairment, executive function, migraine without aura, saccadometry

## Abstract

**Including:**

Migraine without aura is a common neurological disorder associated with structural and functional changes in the brain, which may lead to impairments in cognitive control and motor function. Functional magnetic resonance imaging (fMRI) has demonstrated changes in cortical gray matter volume associated with pain and ocular function in patients with migraine without aura.

**Objective:**

This study aims to evaluate prosaccade and antisaccade eye movements in patients with migraine without aura (MWOA) and determine whether the saccadometry test is an effective tool for identifying cognitive control and executive dysfunctions in these individuals.

**Methods:**

This study included 40 patients diagnosed with migraine without aura (MWOA) and 40 healthy controls. Prosaccade and anti‐saccade eye movements were evaluated using a saccadometry test. The evaluation parameters were the latency, velocity, accuracy, overall error percentage, and directional error percentage.

**Results:**

The MWOA group performed worse on antisaccadic tasks compared to the control group. The MWOA group exhibited elevated overall and directional error rates, prolonged latency, and diminished accuracy (*p* = 0.0001) and velocity (*p* = 0.001) in comparison to the control group. No significant differences were seen in latency, velocity, and accuracy values for prosaccadic movements (*p* > 0.05), however general and directional error rates were significantly elevated (*p* = 0.014).

**Conclusion:**

This study shows that migraine patients without aura experience difficulties in cognitive control and executive functions in antisaccadic eye movement tasks. Although prosaccade reflexive eye movements were generally preserved, significant difficulties were found in directional control and error suppression during the task. Our findings emphasize the potential for saccadometry to be an effective tool for assessing these impairments. The results may contribute to the development of more targeted treatment strategies for MWOA patients.

## Graphical abstract

This study highlights cognitive control and executive dysfunctions in patients with migraine without aura, measured through antisaccadic eye movements. While prosaccadic movements were largely preserved, significant difficulties were observed in the antisaccade test across all parameters. Saccadometry may serve as a potential tool for assessing these impairments and guiding targeted treatments.

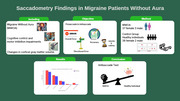

## 1 Introduction

Migraine is a common neurological disorder worldwide and is characterized by headache attacks as well as various neurological and systemic symptoms (Rasmussen et al. 1991). Studies have shown that individuals with migraine have decreased attention, memory, executive functions, and processing speed; these cognitive impairments may be directly related to the frequency and severity of migraine attacks and may negatively affect the patient's quality of life (Gil‐Gouveia and Martins 2019).

Neuroimaging studies indicate that the pathophysiology of migraine leads to significant changes in brain structures (Filippi and Messina, 2020; Goadsby et al., 2002; Pietrobon et al., 2013). In migraine patients, reductions in gray matter volume are particularly concentrated in the insula, motor/premotor, cingulate, prefrontal, and parietal cortices (Kim et al. 2008). These reduced brain volumes are associated not only with headache severity but also with cognitive dysfunction (Hodgson et al. 2019; Pierrot‐Deseilligny et al. 1991). Additionally, functional magnetic resonance imaging (fMRI) has shown alterations in cortical gray matter volume related to ocular function in patients with migraine without aura (Russo et al. 2017; Wilkinson et al. 2006). In migraine patients, these structural changes overlap with areas responsible for controlling saccadic eye movements. Eye movements assessed by saccadometry rely on both motor and executive functions (Hodgson et al. 2019; Pierrot‐Deseilligny et al. 1991). Degeneration in these regions can lead to impairments in executive functions evaluated by antisaccade tasks, potentially contributing to cognitive and motor dysfunctions associated with headaches.

Saccadometry is an advanced oculomotor test that assesses the brain regions that govern saccadic eye movements and their function. The test includes prosaccade (reflexive) and antisaccade analyses. Prosaccades are automatic and rapid eye movements directed towards a visual target (Munoz et al. 2004). They usually do not require conscious control. These movements are controlled by the superior colliculus and brainstem. Antisaccades are associated with more complex executive functions such as cognitive control, emotional regulation and response inhibition. The saccade inhibition task involves executive skills such as consciously stopping target‐directed eye movements and directing the eyes in the opposite direction of the target. This process requires working memory and attention (Coe et al. 2017). Antisaccade movement is triggered by inhibitory signals sent from the dorsolateral prefrontal cortex (DLPFC) to the superior colliculus (SOC) to inhibit reflexive saccades. In migraine patients, functional changes can be seen especially in regions such as DLPFC and SOC, which may lead to impairments in saccadic eye movements (Ptak and Müri 2013; Qin et al. 2020). Antisaccade tests have been used to identify difficulties in inhibiting automatic prosaccades in individuals with neurological or psychiatric disorders affecting brain regions such as the frontal lobes or basal ganglia (Hodgson et al. 2019; Si, Wang, and Zhao 2022).

Previous studies have drawn attention to the increase in antisaccade error rates of migraine patients (Wieser, Wolff, and Hoffmann 2004; Wilkinson et al. 2006). This indicates that migraine patients have difficulties in suppressing reflexive saccadic eye movements. This provides evidence that migraine patients have cognitive impairments especially in executive functions. In accordance with previous neuroimaging studies, it was thought that dysfunctions observed in saccadic eye movements in migraine could be considered as functional reflections of decreases in grey matter volume (Koppen et al. 2011; Russo et al. 2017).

Neuropsychological tests show that cognitive performance is impaired in patients with migraine with aura, especially in verbal and visual memory (Martins et al. 2012; Mickleborough et al. 2011), executive functions (Freitas et al. 2012) (), psychomotor speed (Demarquay et al. 2011; Mickleborough et al. 2011), attention (Demarquay et al. 2011) and language (Wen et al. 2016). However, the presence of such cognitive impairments in patients with migraine without aura (MWOA) is still a matter of debate (Groot et al. n.d.). In this context, saccadometry test provides an important quantitative method for the evaluation of cognitive functions. Especially in recent years, some manufacturers have expanded the practical clinical use of this method by adding saccadometry to vestibular test batteries. It is thought that saccadometry may be an effective method for the evaluation of MWOA patients. However, there are very limited studies with saccadometry in MWOA patients in the literature.

This study aims to evaluate prosaccade and antisaccade eye movements in patients with MWOA and determine whether the saccadometry test is an effective tool for identifying cognitive control and executive dysfunctions in these individuals.

## 2 Material and Methods

This study was conducted in accordance with the ethical guidelines determined by the National Institutes of Health based on the Declaration of Helsinki. The research protocol was approved by the University of Health Sciences Scientific Research Ethics Committee on 22.08.2024 (Decision No: 24/385). All participants agreed to participate in the study by signing the informed consent form.

## 3 Participants

This study was conducted with 40 MWOA patients (37 females, three males; mean age ± SD = 38.5 ± 12) and 40 healthy controls (38 females, two males; mean age ± SD = 30.18 ± 11.2). Participants were recruited from the Neurology Clinic of Ümraniye Training and Research Hospital, Health Sciences University. Patient selection was made from patients who had been followed for at least 5 years and had attacks once or twice a month. The educational level of the participants was at least high school graduate and they were selected from individuals with similar sociocultural level. Neurological examinations and Mini‐Mental State Examination (MMSE) tests were performed by neurology specialists. To assess eligibility criteria, pure‐tone audiometry, speech audiometry, tympanometric evaluation, and acoustic reflex measurements were conducted. Additionally, oculomotor tests were performed to exclude central vestibular system dysfunction. All tests were carried out by research audiologists at the Audiology Center of the same hospital. Participants were instructed not to take any medications that could affect saccadic control prior to the recordings. It was confirmed that migraine patients had not received any preventive treatments within the last three months. The control group consisted of individuals with no history of headache, no neurological disorders or conditions, and no regular use of medication. Subjects with speech discrimination scores below 88% or hearing thresholds above 25 dB HL were excluded from participation in either the patient or control groups.

## 4 Power Analysis

The sample size was calculated using the GPower 3.1 program with a medium effect size of 0.5 and a margin of error of 5% to ensure at least 90% power.

## 5 Exclusion Criteria and Inclusion Criteria

### 5.1 Inclusion Criteria

1. According to IHS Classification ICHD‐3 (International Classification of Headache Disorders, 3rd edition) criteria
  - At least five attacks fulfil the B and D criteria
  - Headache attacks lasting 4–72 h (untreated or unsuccessfully treated)
  - Headache has at least two of the following four characteristics:
    - Unilateral
    - Throbbing pain character
    - Moderate or severe pain intensity
    - Avoidance or worsening of routine physical activity (e.g., walking or climbing stairs)
  - If at least one of the following is present during the headache:
    - Nausea and/or vomiting
    - Photophobia and phonophobia
2. 18–45 years old
3. Ability to co‐operate and complete examinations for intentional saccadic eye movements
4. Patients are required to sign the informed consent form and give their consent (Migraine without Aura‐ ICHD‐3 n.d.)

### 5.2 Exclusion Criteria

Exclusion criteria

‐ Individuals taking medication that affects saccade control (such as selective serotonin reuptake inhibitors and tranquillisers),
‐ Individuals with aphasia, mental and cognitive impairment,
‐ Alzheimer's disease, Parkinson's disease, previous stroke and history,
‐ Individuals experiencing headache during the test
‐ Conditions that may affect the results of saccadic eye movements,
‐ Individuals with hearing loss were not included in the study, and
‐ Cognitive dysfunction was defined as (Mini‐Mental State Examination [MMSE] score ≤24) because it may affect the patient's understanding of instructions (Psikiyatri, and 2018, n.d.).

## 6 Procedures

Pure tone audiometry and speech audiometry were performed in quiet rooms using a clinical audiometer (Madsen Astera; Denmark) of IAC (Industrial Acoustic Company) standards. Air conduction hearing thresholds were measured in the range of 125–8000 Hz.

Tympanometric evaluation (with 226 Hz probe tone) and both stapedial reflex measurements were performed using a tympanometer (Madsen Otoflex 100; Denmark).

Oculomotor tests (spontaneous nystagmus, gaze, smooth pursuit, random saccades, optokinetic nystagmus tests in the VNG test battery) were performed using a VNG (Interacoustics VisualEyes 525 Denmark).

## 7 Saccadometry

The saccadometry test was performed in a separate session from the tests that determined the inclusion criteria.

Saccadometry tests were performed using a VNG (Interacoustics VisualEyes 525; Denmark). The manufacturer's standard settings were used for the test parameters. The individual was seated in a fixed chair 1.2 m away from the screen. For each test, a red stimulus target was presented on a black background on the TV screen.

The stimulus target was 1% of the total width of the screen. Each stimulus target was presented on the screen at a random distance of 10° to the left or right of the fixed center target after a random delay (1–2 s; mean interval duration 1.5 s). The test was performed in the horizontal plane. VOG goggles were worn and calibrated at the beginning of the test to record eye movements. To minimize response‐related artefacts, each patient was instructed to keep his/her head still, eyes open and follow the instructions given.

In the prosaccade test, a fixed dot lights up in the center and another dot flashes randomly. The participant was asked to look at the randomly lit dot on the screen and then look again at the fixed dot lit in the center.

In the antisaccade test, the participant was first told to look at a fixed central point. When a new light appeared, the participant was asked to look away from it and then return to the central target.

The test was performed in the test procedure as a 100 jumps/50 right, 50 left (251 ms) block trial. Block trial refers to the same test parameters (only prosaccades or only antisaccades) performed within one trial (Antoniades, Ettinger, and Gaymard 2013). At the end of the test, the following parameters calculated by the software were evaluated:

Peak velocity (degrees per second): It shows the movement speed of the eyes between two points.

Delay (ms): It is the time from the time the stimulus moves until the eye movement is initiated.

Accuracy (percentage of actual eye movement and target eye movement): Indicates the ability to move the eyes directly toward the target.

Direction error rate: It is the percentage of the patient's eyes moving in the wrong direction (Antoniades, Ettinger, and Gaymard 2013).

**FIGURE 1:**
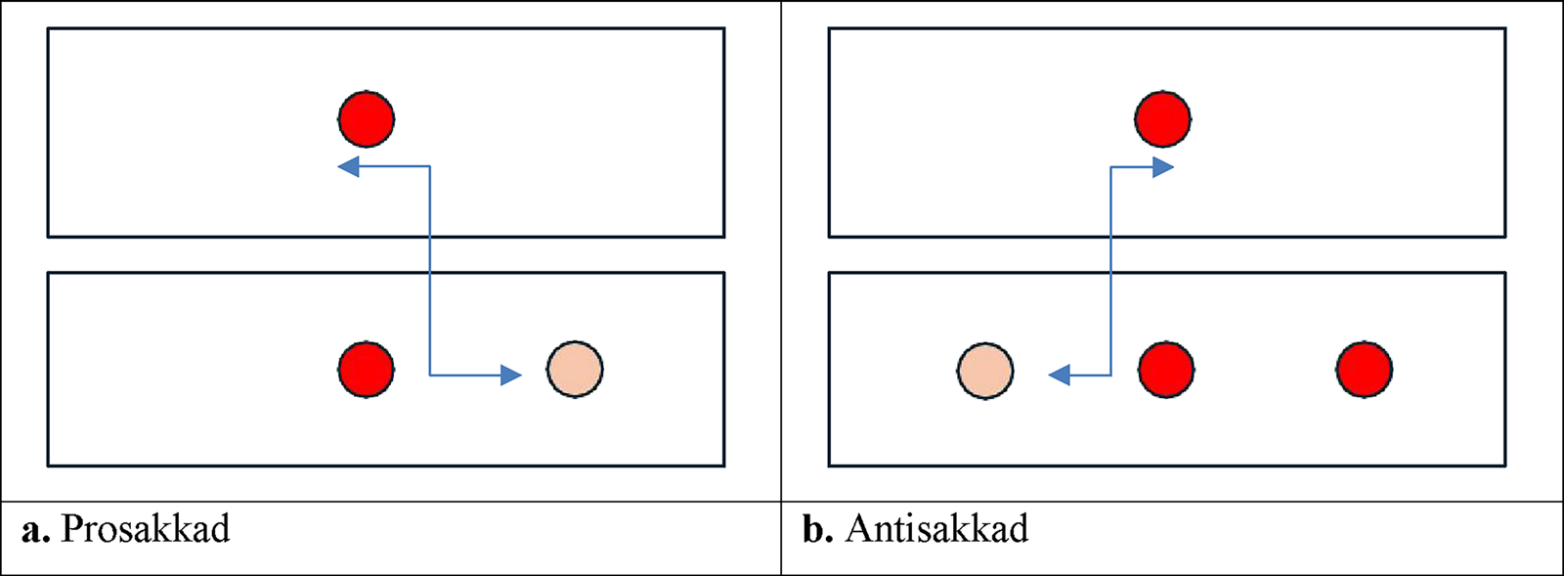
The dark red dot is the fixed point lit in the center. The light‐colored dot is the target point expected from the subject. In the prosaccade test, the subject is asked to look first at the central point, then at the target point and then at the central point again. In the antisaccade test, the subject is asked to look at the central dot and then look at the space where the target dot is located in its symmetry without looking at the second red dot.

For the above parameters in saccadometry test, separate and average values can be obtained for the right and left eyes. In this study, the mean values in both target directions for both eyes were statistically analyzed. The tests were performed by the same experienced clinician to eliminate clinician‐dependent variability. Subjects were asked about conditions such as illness, fatigue, and insomnia that may be related to mental fatigue (Ishii et al. 2014). These conditions were excluded to perform the saccadometry test under the same conditions. The image shows a diagram of antisaccade and prosaccade movements (Figure 1).

## 8 Statistical Analysis

All statistical analyses were performed using IBM SPSS Statistics Version 26. Descriptive statistics were calculated for the demographic data of the migraine and control groups, as well as for the measurements related to prosaccade and antisaccade tasks. These descriptive statistics included the median, interquartile range (IQR), minimum, maximum, mean, and standard deviation. To determine the appropriate statistical tests, the normality of the variables in both groups was assessed using the Shapiro–Wilk test. For variables showing normal distribution, the Independent Samples *t*‐test was used. For variables not showing normal distribution, the Mann–Whitney *U* test was applied. Comparisons of demographic data and measurements for prosaccade and antisaccade tasks were presented in the relevant tables. All statistical analyses were conducted with a 95% confidence interval, and a significance level of *p* < 0.05 was considered.

## 9 Results

The comparison of the demographic data of the participants is shown in Table 1.

**TABLE 1:** Comparison of demographic data of the participants.

Demographic data	MWOA *n *= 40	Control group *n *= 40	*p*
	Ort. ± SS veya *n* (%)	Ort.±SS veya *n* (%)	
Age	38.5 ± 12	30.18 ± 11.2	0.001^b^
Gender			
Female	37 (92.5)	38 (70)	0.01^a^
Male	3 (7.5)	2 (30)	

*Note*: *p* < 0.05.

^a^ Mann–Whitney U test.

^b^ Chi‐square test.

The mean age of the migraine group was 38.5 ± 12, while the mean age of the control group was 30.18 ± 11.2. There is a statistically significant difference between the groups in terms of age (Mann–Whitney *U* test; *U* = 469.5; *Z* = −3,183; *p* = 0.001). In the migraine group, 37 (92.5%) were female and 3 (7.5%) were male; in the control group, 38 (95%) were female and 2 (5%) were male. There was no statistically significant difference between the groups in terms of gender (Chi‐square test; X^2^ = 0.6; *p* = 0.01).

Prosaccade latency, speed, accuracy, general error and directional error findings between migraine without aura and control group are shown in Table 2.

**TABLE 2:** Prosaccade findings between migraine without aura and control group.

Prosaccade	MWOA (*n *= 40)			Control group (*n* = 40)			*p*
	Median (IQR)	Min–Max	Ort. ± SS	Median (IQR)	Min–Max	Ort. ± SS	
Mean latency (ms)	240.5 (68)	27–347	239.78 ± 53.2	251.5 (39)	193–310	246.93 ± 26	0.513
Mean velocity (°/s)	277 (27)	227–344	274.9 ± 21	279.5 (23)	238–311	275.18 ± 18.7	0.78
Mean accuracy (%)	98 (5)	84–115	97.88 ± 5.7	97.5 (6)	86–105	96.55 ± 5	0.44
Directional error rate (%)	0 (1)	0–5	0.88 ± 1.3	0 (0)	0–3	0.3 ± 0.6	0.014
Overall error rate (%)	10.5 (12)	2–30	12.25 ± 7.3	3 (3)	0–13	3.6 ± 3.3	0.0001

*Note*: Mann–Whitney U test; *p* < 0.05.

There is no statistically significant difference between the groups in terms of prosaccade mean latency, speed and accuracy values. There is a statistically significant difference between the groups in terms of prosaccade directional error (%) (Mann–Whitney *U* test; *U* = 584; *Z* = −2.464; *p* = 0,014). There is a statistically significant difference between the groups in terms of prosaccade general error (%) (Mann–Whitney *U* test; *U* = 189; *Z* = −5.897; *p* = 0.0001).

Antisaccade latency, speed, accuracy, general error and directional error findings between migraine without aura and control group are shown in Table 3.

**TABLE 3:** Antisaccade findings between migraine without aura and control group.

Antisaccade	MWOA (*n *= 40)			Control group (*n *= 40)			*p*
	Median (IQR)	Min–Max	Ort. ± SS	Median (IQR)	Min–Max	Ort. ± SS	
Mean latency (ms)	368.5 (59)	267–522	382.05 ± 53.3	331.5 (49)	247–425	332.7 ± 39.6	0.0001^b^
Mean velocity(^0^/s)	244 (61)	180–356	254.83 ± 44.4	217.5 (57)	169–357	223.8 ± 37.1	0.001^a^
Mean accuracy (%)	98.5 (33)	69–214	107.05 ± 31.6	83 (21)	57–136	85.2 ± 18.4	0.0001^a^
Directional error rate (%)	17 (14)	3–53	20.75 ± 11.8	5 (5)	0–19	5.3 ± 3.9	0.0001^a^
Overall error rate (%)	43.5 (31)	11–69	40.13 ± 16.3	9 (8)	2–28	10.5 ± 5.8	0.0001^a^

*Note*: *p* < 0.05.

^a^Mann–Whitney U test.

^b^Independent samples *t* test.

There is a statistically significant difference between the groups in terms of antisaccade mean latency (Independent samples *t* test; *t* = 4.7; *p* = 0.0001). There is a statistically significant difference between the groups in terms of average speed (Mann–Whitney *U* test; *U* = 466; *Z* = −3.215; *p* = 0,001). There is a statistically significant difference between the groups in terms of mean accuracy (Mann–Whitney *U* test; *U* = 405.5; *Z* = −3,798; *p* = 0.0001). There is a statistically significant difference between the groups in terms of directional error (%) (Mann–Whitney *U* test; *U* = 105; *Z* = −6.701; *p* = 0.0001). There is a statistically significant difference between the groups in terms of general error (%) (Mann–Whitney *U* test; *U* = 65; *Z* = −7.079; *p* = 0,0001).

## 10 Discussion

The aim of this study was to evaluate prosaccade and antisaccade eye movements in migraine patients without aura (MWOA) and to determine the effectiveness of the saccadometry test in detecting cognitive control and executive dysfunction in these patients. The MWOA group performed worse than the control group in terms of latency, velocity, directional error rate and overall error rate in antisaccadic tasks. Significant differences were noted in prosaccade parameters, directional error rates and overall error rates, but no statistical differences were found in mean latency, velocity or accuracy values.

Prosaccade eye movements are reflexive eye movements that occur due to rapid perception of visual stimuli. These movements are regulated by neural coordination between the basal ganglia (BG), superior colliculus (SC), brainstem, and cortical areas. Especially the basal ganglia plays a key role in the initiation and stopping of these movements (Hikosaka and Wurtz 1983; Leigh and Zee 2015). The brainstem and frontal eye fields (FEF) enable rapid response to visual targets, while the cerebellum regulates the accuracy and speed of movements. Coordination between these neural structures ensures that prosaccadic movements are an efficient and effective response to environmental stimuli (Leigh and Zee 2015). There are many studies on saccadic eye movements in migraine patients. Cambron et al. (2011) and Harno et al. (2003) showed that there was a significant difference in the migraine group compared to normal controls. In another study conducted by Filippopulos et al. in patients with migraine, it was found that the function of reflexive saccades was not affected (Filippopulos et al. 2021). Studies show that recurrent headache, especially migraine attacks, may lead to lesions in the white matter. These lesions are structural changes observed in the brain in migraine patients. These changes have been found to be more common in patients with long‐term, frequently recurrent migraine attacks (Filippopulos et al. 2021). In our study, a significant difference was found in the directional error and general error rates in the migraine patient group compared to the control groups. These findings are consistent with the results of Zhaoxia Qin et al. which revealed that functional connections between sensorimotor areas and other brain regions were impaired in MWOA individuals. In addition, it has been reported that impairment in these connections may have negative effects on motor control and cognitive functions (Qin et al. 2020). In our study, poor performance in direction error rates may suggest weakened coordination between these structures in migraine patients. Our findings indicate that reflexive eye movements are generally preserved in individuals with MWOA; however, there may be issues with direction control and error suppression processes.

The antisaccade mechanism is a more complex process compared to prosaccades and reflects the cognitive control abilities of the brain. Antisaccade eye movements are voluntary movements in the opposite direction of a visual stimulus and this process is possible by inhibiting the prosaccade response (Coe et al. 2017). DLPFC, anterior cingulate cortex (ACC) and BG play an important role in this process. DLPFC has been reported to be especially important in the control of attention and inhibition (Aron, Robbins, and Poldrack 2014; Coe et al. 2017). Various studies have revealed that DLPFC lesions increase the likelihood of involuntary eye movements towards the target in antisaccade tasks. In addition, frontal eye field (FEF) lesions cause delayed onset of eye movements despite saccades in the correct direction. It has been emphasized that FEF plays a critical role in regulating the timing of eye movements and DLPFC plays a critical role in suppressing involuntary movements (Butler, Zacks, and Henderson 1999; Pierrot‐Deseilligny et al. 2005). Cambron et al. found that migraine patients made more errors in antisaccade tasks and showed longer latency periods (Cambron et al. 2011). Filippopulos et al. similarly reported prolongation in saccade latencies only in the antisaccade task in patients with and without aura compared to the control group (Filippopulos et al. 2021). These findings point to FEF and DLPFC dysfunctions (Qin et al. 2020). The increased error rates observed in migraine patients, especially in these tasks in which attention, inhibition and executive functions are negatively affected, may also be related to grey matter volume losses in the brain (Cambron et al. 2011). Compared to the control group, migraine patients' significantly lower percentage of valid saccades in antisaccade tasks may indicate that their ability to initiate deliberate saccades is impaired. Furthermore, damage to the FEF may increase the latency of voluntary saccades, which would explain the increased latency durations observed in migraine patients.

It has been reported in many studies that cognitive functions are weakened in individuals with migraine especially difficulty in directional control and error suppression (Chong et al. 2017; Gu et al. 2022). These cognitive deficits may manifest themselves in tasks requiring cognitive control such as saccadic eye movements. A meta‐analysis study by Lihua Gu et al. examining the relationship between migraine and cognitive impairment revealed that individuals with migraine exhibit lower performance in general cognitive function and language functions (Gu et al. 2022). These results are in parallel with the findings in the literature regarding the complex effects of migraine on cognitive processes. In addition, this analysis also emphasized that migraine is associated with an increased risk of dementia, vascular dementia and Alzheimer's disease (Gu et al. 2022). In the study conducted by Gabriella et al., it was found that migraine patients without aura exhibited mild cognitive dysfunctions and low rates of behavioral symptoms. The researchers used the MoCA (Montreal Cognitive Assessment) scale in this study. It was found that migraine patients without aura had significantly lower scores in executive function, attention, visuospatial and memory domains of the scale compared to the control group (Santangelo et al. 2016). This finding aligns with the low performance observed in antisaccadic tasks in our study and suggests that individuals with migraines may experience some challenges in executive functions and cognitive processes. The results indicate that attention and inhibition functions assessed through antisaccadic tasks might be negatively affected in individuals with MWOA.

The findings in the literature provide important clues to make sense of the poor performance of individuals with migraine in all parameters in antisaccade tasks in our study. Poor performance in antisaccade tasks may provide evidence that it is related to difficulties in cognitive control functions in these individuals. We also suggest that poor performance may be a reflection of difficulties in directional control and error suppression.

## 11 Conclusion

In our study, it was observed that individuals with migraine without aura exhibited differences in prosaccadic and antisaccadic eye movement performance compared to the control group. While overall performance in prosaccadic eye movements appeared to be largely preserved, increases in directional error and overall error rates suggest that migraineurs may experience some challenges in motor control processes. In antisaccadic tasks, migraineurs demonstrated higher error rates and longer latencies compared to healthy individuals, which may be associated with difficulties in attention inhibition and executive functions. The cognitive challenges and neurological effects associated with migraine reported in the literature align with the findings of our study. These results suggest that migraine is not solely limited to headaches but may also have effects on cognitive and neurological processes.

## 12 Limitations

This study is limited to patients with migraine without aura, which restricts the generalizability of the findings to migraines with aura and other types of headaches. Future studies examining antisaccadic performance in different headache types could contribute to a better understanding of the neurophysiological and cognitive mechanisms underlying these conditions. Additionally, supporting the findings with methods such as fMRI would enable a more in‐depth investigation of the related mechanisms.

Our sample predominantly consists of female participants, which limits the evaluation of gender differences. Future studies could ensure a more balanced sample in terms of participants' educational background, age, and gender. Furthermore, longitudinal studies monitoring changes in antisaccadic performance over time may provide a better understanding of the relationship between these parameters and cognitive functions.

## Author Contributions

**Asya Fatma Men**: conceptualization, investigation, funding acquisition, writing – original draft, methodology, validation, visualization, writing – review and editing, resources, project administration, formal analysis. **Aysenur Kucuk Ceyhan**: writing – review and editing. **Mustafa Men**: Validation, resources, writing – review and editing, methodology. **Reyhan Sürmeli**: methodology, validation, writing – review and editing, resources.

### Peer Review

The peer review history for this article is available at https://publons.com/publon/10.1002/brb3.70367.

## Ethics Statement

The research protocol was approved by the University of Health Sciences Scientific Research Ethics Committee on 22.08.2024 (Decision No: 24/385).

## Data Availability

The data that support the findings of this study are available from the corresponding author upon reasonable request.
